# Cholesterol Efflux Efficiency of Reconstituted HDL Is Affected by Nanoparticle Lipid Composition

**DOI:** 10.3390/biomedicines8100373

**Published:** 2020-09-23

**Authors:** Shifa Jebari-Benslaiman, Kepa B. Uribe, Asier Benito-Vicente, Unai Galicia-Garcia, Asier Larrea-Sebal, Iraide Alloza, Koen Vandenbroeck, Helena Ostolaza, César Martín

**Affiliations:** 1 Department of Molecular Biophysics, Biofisika Institute (University of Basque Country and Consejo Superior de Investigaciones Científicas (UPV/EHU, CSIC)), 48940 Leioa, Spain; shifajebari@gmail.com (S.J.-B.); asierbenitovicente@gmail.com (A.B.-V.); u.galiciag@gmail.com (U.G.-G.); asierlarrea@yahoo.es (A.L.-S.); elenaamaya.ostolaza@ehu.eus (H.O.); 2 Department of Biochemistry and Molecular Biology, University of the Basque Country (UPV/EHU), 48940 Leioa, Spain; iraide.allozamoral@osakidetza.eus (I.A.); k.vandenbroeck@ikerbasque.org (K.V.); 3 Center for Cooperative Research in Biomaterials (CIC biomaGUNE), Basque Research and Technology Alliance (BRTA), 20018 Donostia San Sebastián, Spain; kbelloso@cicbiomagune.es; 4 Department of Molecular Biophysics, Fundación Biofísica Bizkaia, 48940 Leioa, Spain; 5 Inflammation & Biomarkers Group, Biocruces Bizkaia Health Research Institute, 48903 Barakaldo, Spain; 6 Ikerbasque, Basque Foundation for Science, 48013 Bilbao, Spain

**Keywords:** apoA-I, rHDL, nanodisc, cholesterol efflux, cardiovascular disease

## Abstract

Cardiovascular disease (CVD), the leading cause of mortality worldwide is primarily caused by atherosclerosis, which is promoted by the accumulation of low-density lipoproteins into the intima of large arteries. Multiple nanoparticles mimicking natural HDL (rHDL) have been designed to remove cholesterol excess in CVD therapy. The goal of this investigation was to assess the cholesterol efflux efficiency of rHDLs with different lipid compositions, mimicking different maturation stages of high-density lipoproteins (HDLs) occurring in vivo. Methods: the cholesterol efflux activity of soybean PC (Soy-PC), 1,2-dipalmitoyl-sn-glycero-3-phosphocholine (DPPC), DPPC:Chol:1-palmitoyl-2-hydroxy-sn-glycero-3-phosphocholine (LysoPC) and DPPC:18:2 cholesteryl ester (CE):LysoPC rHDLs was determined in several cell models to investigate the contribution of lipid composition to the effectiveness of cholesterol removal. Results: DPPC rHDLs are the most efficient particles, inducing cholesterol efflux in all cellular models and in all conditions the effect was potentiated when the ABCA1 transporter was upregulated. Conclusions: DPPC rHDLs, which resemble nascent HDL, are the most effective particles in inducing cholesterol efflux due to the higher physical binding affinity of cholesterol to the saturated long-chain-length phospholipids and the favored cholesterol transfer from a highly positively curved bilayer, to an accepting planar bilayer such as DPPC rHDLs. The physicochemical characteristics of rHDLs should be taken into consideration to design more efficient nanoparticles to promote cholesterol efflux.

## 1. Introduction

Cardiovascular disease (CVD), the leading cause of mortality in industrially developed countries [1], is primarily caused by atherosclerosis, characterized by an abnormal lipid and inflammatory cell accumulation in the intima, the subendothelial layer of large arteries [2]. Atherosclerosis is associated with atheroma plaque formation and reduction in the vascular diameter, thus increasing the incidence of cardiovascular events [3]. Atherogenesis is initiated and maintained by selective entrapment of low-density lipoproteins (LDL) in the extracellular arterial intima, and is mediated by the interaction of specific positively charged amino acyl residues of apoB100 with arterial proteoglycans [4,5,6]. This interaction constitutes the central biochemical and pathogenic process because the proteoglycans initiate lipoprotein degradation with the production of bioactive, lipid products that trigger an inflammatory response which leads to aterosclerosis [7]. Within the intima of the artery, LDLs can be modified either by oxidation and/or enzymatic mechanisms and by an inflammatory component [8,9]. The non-regulated uptake of modified LDL particles, mediated by scavenger receptors, promotes cholesterol overloading in macrophages and smooth muscle cells [10] which drives atherosclerotic plaque progression and the activation of inflammatory pathways [11]. Removal of cholesterol excess from peripheral tissues is carried out by apolipoprotein A-I (apoA-I) containing high-density lipoproteins (HDL), through a mechanism known as reverse cholesterol transport (RCT) [12]. This process is facilitated by apoA-I interaction with members of the ATP-binding cassette transporters superfamily such as ABCA1, a cholesterol efflux mediator present in various cell types such as hepatocytes, enterocytes, and macrophages [13,14]. ABCA1 transfers cholesterol to lipid-poor apoA-I and promotes the formation of discoidal particles, denominated nascent HDLs, that are mainly composed of phosphatidylcholine and cholesterol [15,16,17,18,19,20]. In contrast, the ABCG1 transporter mediates cholesterol transport to the assembled HDL [21,22], but not to lipid-free apoA-I [21,23].

Nascent HDL particles are mainly composed of phosphatidylcholine and cholesterol [20], while native mature HDL particles show high levels of structural and compositional heterogeneity, with a quantitative predomination of phospholipids that together with sphingomyelin account for 40–60% of the total lipids, with lesser proportions of cholesteryl esters (30–40%), triglycerides (5–12%), and free colesterol (5–10%) [24].

The interaction of HDLs with membrane cholesterol transporters is especially relevant in reducing the intracellular cholesterol content of macrophages that interact with the atheroma plaque. Reducing macrophage intracellular cholesterol content can avoid macrophage conversion into foam cells, a process that normally promotes atherosclerosis development [25]. In addition, HDLs possess anti-inflammatory, anti-thrombotic and anti-oxidant properties, as well as the ability to support endothelial physiology [26,27].

The inversely associated relationship between low HDL cholesterol levels and CVD risk in epidemiologic studies [28,29] has focused attention on HDL mimetics as a potential therapeutic tool and as an inspirational source for biomedical engineering. Several nanoparticles mimicking nascent HDL features have been designed and used in several clinical trials for CVD therapy [30,31]. These include the following: (1) nanoparticles containing apoA-I Milano known as ETC216 or MDCO216; (2) a discoidal pre-β HDL composed of recombinant human apoA-I and a lipid mixture, denominated CER-001; and finally (3) reconstituted HDL (rHDL) particles consisting of human plasma derived apoA-I and soybean phosphatidylcholine, known as CSL-112. These three formulations, designed to optimize cholesterol efflux by ABCA1, are the most studied nascent HDL mimicking nanoparticles. However, these nanoparticles have yielded markedly different results when tested in vivo. Among them, the capacity of CSL-112 to enhance cholesterol efflux capacity has been confirmed and its effectiveness in reducing major cardiovascular events is being assessed in a Phase III clinical trial [31].

Initially, infusion of apoA-I Milano, a naturally occurring mutation of apoA-I associated with low prevalence of CVD [32,33], showed regression of coronary atherosclerosis in a phase II trial (“The ApoA-I Milano Trial”) [34]. Accordingly, apoA-I Milano was considered as a novel therapeutic strategy to favor reverse-cholesterol transport. However, failure to induce plaque regression in subsequent clinical trials led to discarding this formulation as a therapeutic drug [35].

The next HDL mimetic to be tested as a cellular-sterol efflux inducer was CER-001. This nanoparticle showed the ability to rapidly mobilize large amounts of cholesterol into the HDL fraction; however, the mimetic did not cause any significant reduction in coronary atherosclerosis as evaluated in the CHI-SQUARE study [36]. Interestingly, posterior analyzes showed a *U*-shaped CER-001 dose-response curve with the greatest atheroma regression occurring at a low concentration, while higher concentrations are inefficient in removing cholesterol due to the strong down regulation of the ABCA1 transporter [37].

Finally, the CSL-112 reconstituted HDLs arose as an improvement upon their predecessor, CSL-111. CSL-111 initially showed a potential therapeutic effect [38], but was disfavored due to its hepatotoxicity. On the contrary, CSL-112 was well tolerated and not associated with any significant alterations in liver or kidney function [31]. Moreover, CSL-112 has been found to enhance cholesterol efflux very efficiently. However, its beneficial potential in reducing major adverse cardiovascular events in a group of high-risk patients will be assessed in the on-going large phase III AEGIS-II study (NCT03473223).

Several attempts have been made to assess the role of both protein components (apoA-I, apoA-I mutants, and mimetic peptides) and different phospholipid (PL) compositions in the efficiency of cholesterol efflux induced by reconstituted HDL (rHDL) [34,38,39,40,41,42,43,44]. The goal of the present work was to assess the efficiency of rHDLs with different lipid compositions mimicking different physiologic maturation stages of HDLs. To do so, cholesterol efflux efficiency of soybean PC (Soy-PC), 1,2-dipalmitoyl-sn-glycero-3-phosphocholine (DPPC), DPPC:Chol:1-palmitoyl-2-hydroxy-sn-glycero-3-phosphocholine (LysoPC) and DPPC:CE:LysoPC rHDL with similar sizes was determined in THP-1 and J774A.1 macrophages, J774A.1-derived foam cells and vascular smooth foam cells obtained from human carotid endarterectomy. In addition, we sought to investigate the contribution of lipid composition to the effectiveness of the nanoparticles in cholesterol removal.

## 2. Experimental Section

### 2.1. Materials

Soybean PC (Soy-PC) 1,2-dipalmitoyl-sn-glycero-3-phosphocholine (DPPC), 1-palmitoyl-2-hydroxy-sn-glycero-3-phosphocholine (LysoPC), cholesterol (Chol), 23-(dipyrrometheneboron difluoride)-24-norcholesterol (TopFluor^®^ Cholesterol), were purchased from Avanti Polar Lipids (Alabaster, AL, USA).

Cholesteryl linoleate (18:2 cholesteryl ester, CE), 1,6-diphenyl-1,3,5-hexatriene (DPH), sodium cholate hydrate, Methyl-β-cyclodextrin, heparin, newborn calf serum (NCS), bovine serum albumin (BSA), BSA fatty acid free, Acyl-CoA:cholesterol acyltransferase (ACAT) inhibitor (Sandoz 58-035), T0901317 (TO90) and cell culture reagents were obtained from Sigma-Aldrich (Madrid, Spain). Collagenase type I (ColI, 17018029, Thermo Fisher Scientific, Waltham, MA, USA). Basic fibroblast growth factor (FGFb), insulin-like growth factor 1 (IGF-1) and epidermal growth factor (EGF) were purchased from Miltenyi Biotec (Madrid, España). The synthetic LXR ligand T0901217 (T090) was purchased from Cayman Chemical (Ann Arbor, Michigan, USA).

### 2.2. Human ApoA-I Purification

Human apoA-I was purified from *E. coli* BL21 (DE3) pLysS transformed with apoA-I containing pET32-E43C vector (kindly provided by Prof. Oda, Children’s Hospital Oakland Research Institute (Oakland, CA, USA) as described before [45]. Transformed *E. coli* BL21 (DE3) pLysS were grown in LB medium supplemented with ampicillin (0.1 mg/mL) and chloramphenicol (0.025 mg/mL) at 37 °C. Protein expression was induced with isopropyl β-d-thiogalactopyranoside (IPTG, final concentration 0.4 mM). Bacteria culture was maintained for 3 h at 37 °C. Cells were then harvested and centrifugated at 6000× *g* for 15 min at 4 °C. Bacteria pellet was resuspended in protein extraction buffer (20 mM Tris-HCl, pH 8.0) supplemented with 0.1% (*v*/*v*) IGEPAL CA-630 and protease inhibitors (Complete EDTA-free and PMSF 1:100 (*v*/*v*)). Samples were stored at −80 °C until use.

Samples were then sonicated at 4 °C and cell debris was removed by centrifugation at 9500× *g* for 30 min at 4 °C. Supernatant was filtered through 0.2 µm filters and diluted with loading buffer 1:1 (*v*/*v*) (40 mM NaPO_4_, 1 M NaCl, 6 M Gdn-HCl, pH 7.4). Supernatant was added to a HiTrap TALON^®^ crude 5 mL (GE Healthcare, Chicago, IL, USA), previously equilibrated with 1:2 diluted loading buffer (20 mM NaPO_4_, 500 mM NaCl, 3 M Gdn-HCl, pH 7.4) for 1 h. Then, supernatant was washed with loading buffer first and then with washing buffer (20 mM NaPO_4_, 500 mM NaCl, 20 mM imidazole, pH 7.4). Endotoxin was removed by adding washing buffer with Triton X-114 0.1% (*v*/*v*) and then washed again with washing buffer. Protein was eluted with 20 mM NaPO_4_, 500 mM NaCl, 500 mM imidazole, pH 7.4.

Protein purity was first tested by SDS-PAGE and then dialyzed three times over 24 h, against 20 mM Tris-HCl, 150 mM NaCl, 1 mM EDTA, 1 mM benzamidine hydrochloride, pH 8.0 buffer. Finally, protein aggregates were removed and 10% glycerol was added for protein storage. Concentration of apoA-I was determined from its extinction coefficient at 280 nm, 32,430 M^−1^·cm^−1^.

### 2.3. Human ApoA-I HDL Reconstitution and Purification

Purified apoA-I was incubated with different lipids in a molar ratio of apoA-I to lipids of 1:125 (mol/mol). The lipid compositions used were Soy-PC, DPPC, DPPC:Chol:lysoPC (85:10:5% mol) and DPPC:CE:lysoPC (75:20:5% mol). First, lipid mixtures in chloroform:methanol (2:1 *v*/*v*) were dried with a stream of nitrogen followed by vacuum drying for 1.5–2 h. Lipids were then resuspended in TEN buffer (10 mM Tris-HCl, 1 mM EDTA, 150 mM NaCl, pH 8.0) at 42 °C and sodium cholate was added in a molar ratio of 1:1.2 (total lipid:cholate). Next, apoA-I was added and the resulting solution was incubated above lipid transition temperature (T_m_) for 15 min and mixed vigorously every 5 min. The samples were then incubated overnight in agitation at the same temperature and subsequently dialyzed at 42 °C during 48 h against TEN buffer to remove the cholate.

rHDL purification was performed on a Superdex 200 10/300 GL (GE Healthcare) with TEN buffer as eluent. The column was first calibrated with molecular size standards from Amersham Biosciences. Samples were eluted at 4 °C at a 0.2 mL/min flow rate and elution profiles were expressed as retention volume.

### 2.4. rHDL Biophysical Characterization

#### 2.4.1. Circular Dichroism

The secondary structure of apoA-I was analyzed in a thermostatized Jasco 810 spectropolarimeter in a 0.1 cm path length quartz cuvette. Spectra (200 to 260 nm) were obtained at 25 °C with a bandwidth of 1 nm, response time 1 s and 50 nm/scan speed. Each spectrum represents the average of 15 accumulations and was corrected subtracting the buffer spectra. The α-helicity of the protein for each rHDL composition was calculated using the mean residue ellipticity at 222 nm using the following equation: % α-helix = ((θ)_222_ + 3000)/(36000 + 3000) × 100 [46].

#### 2.4.2. Dynamic Light Scattering (DLS)

The size of the rHDL was determined by dynamic light scattering in a Nano-S Zetasizer (Malvern Instruments, Malvern, UK) as previously described [47]. Measurements were performed in triplicate (15 runs) at 25 °C. Viscosity and refractive index of TEN buffer were applied to the measurements.

#### 2.4.3. Negative Stain Electron Microscopy (NS-EM)

Size and morphology of the rHDL were determined by adsorbing 10 μL of each rHDL preparation on a glow-discharged thin carbon-coated 300-mesh copper grid (Cu-300CN; Pacific Grid-Tech, San Francisco, CA, USA) as previously described [48]. The excess of solution was removed and the grid was washed three times in deionized water. Then, one drop (~30 μL) of 1% (*w*/*v*) uranyl acetate (UA) (pH 4.6) solution was applied and maintained for 1–3 min in the dark before excess stain was removed. Finally, the excess of solution was removed and the sample was air dried at room temperature.

Feret diameter was used to determine particle size by automatically selecting individual particles and then checked to remove overlapping or damaged particles. 1600 particle images from micrographs of each rHDL sample were used for the statistical analysis of particle size distribution.

#### 2.4.4. rHDLs Transition Temperature: Steady State Fluorescence Measurements

Transition temperature of rHDL-lipid moiety was assessed by fluorescence anisotropy, which allows one to follow changes in the order of lipid bilayers taking advantage of the differences in the fluorescence polarization caused by orientation changes of a fluorophore in space. Briefly, the basis of the technique relies on the dependence of absorption and the emission of light on the orientation of the transition dipole moments. Hence, excitation with vertical polarized light can provide information on the rotational motion of a fluorophore because the emitted light will retain some of that polarization based on how fast it is rotating in solution. The extent of depolarization of the emission of a fluorophore in a lipid membrane reflects the degree to which a population of photoselected excited fluorophores loses its initial selective orientation and becomes randomized. Several studies revealed that anisotropy is mainly determined by the degree to which the fluorophore rotations are restricted by the molecular packing of the lipids [49,50].

The order of lipid bilayers in the different rHDLs was analyzed by measuring fluorescence anisotropy of diphenylhexatriene (DPH) [51]. DPH is a fluorescent probe that locates at the lipid-water interface in lipid bilayers and its fluorescence anisotropy is highly dependent on the lipid phase state, which decreases abruptly during the thermotropic gel-fluid phase transition.

DPH in methanol was added to nanoparticles in a final molar ratio of 1:75 (probe to lipid) and the mixture was incubated 1 h in agitation at 25 °C to incorporate DPH into the lipid bilayer of the nanoparticles.

Fluorescence anisotropy (*r*) of DPH was measured (λex: 360 nm, λem: 428) on a SFM25 spectrofluorimeter (Kontron Instruments, Regensdorf, Switzerland) increasing temperature from 26 °C to 60 °C, in increments of 2 °C.

Fluorescence anisotropy was determined as:

$$r\;(t)\;=\;\frac{I_{VV}\left(t\right)-\;GI_{VH}\left(t\right)}{I_{VV}\left(t\right)+\;2GI_{VH}\left(t\right)}$$

where *I_VV_* and *I_VH_* are the intensities of vertically and horizontally polarized fluorescent light, respectively, when the excitation light is vertically polarized. G represents the compensating factor for the anisotropy sensitivity of the instrument, which is expressed as follows:

$$G\;=\;\frac{I_{HV}}{I_{HH}}$$

where *I_HV_* and *I_HH_* refer to vertically and horizontally polarized light intensities, respectively, when excitation light is horizontally polarized.

### 2.5. Isolation of Human Plasma HDL and LDL

Human HDLs and LDLs were isolated from the human plasma of healthy individuals by ultracentrifugation as previously described [52]. Briefly, blood was collected in EDTA tubes and samples were centrifuged for 10 min at 3000× *g* and at 4 °C. Then, plasma density was adjusted to 1.4 g/mL using KBr and layered with cold PBS buffer, pH 7.4 to obtain two phases. Finally, samples were centrifuged at 27,000 rpm for 22 h at 4 °C and the bands corresponding to HDLs and LDLs were recovered carefully and stored at 4 °C until use.

### 2.6. LDL Acetylation

Human LDL acetylation was achieved by mixing 1 mL of concentrated LDLs in PBS with 1 mL of saturated sodium acetate solution under constant stirring at 4 °C, as previously described [53]. Little aliquots of acetic anhydride were added during an hour to get a 40 molar excess of acetic anhydride to lysine content of LDLs. The LDL solution was stirred for an additional 30 min and then dialysed with 12 L of PBS containing 0.3 mM EDTA, pH 7.4 for 24 h. Acetylation was confirmed by agarose gel electrophoresis.

### 2.7. Cell Cultures

Human THP-1 monocytes and murine J774A.1 macrophages cell lines were obtained from ATCC (American Type Culture Collection, Manassas, Virginia, USA). THP-1 were cultured in RPMI-1640 supplemented with 10% FBS (*v*/*v*), 100 µg/mL streptomycin, 100 U/mL penicillin, l-glutamine and MycoZap^TM^ Prophylactic and differentiated into macrophages (2.5 × 10^5^ cells/well in a 24-well plate) with 100 nM phorbol 12-myristate 13-acetate (PMA) for 24 h and maintained in fresh media for the following 72 h.

J774A.1 macrophages were cultured in Dulbecco’s modified Eagles Medium (DMEM, low glucose) supplemented with 10% FBS (*v*/*v*), 100 µg/mL streptomycin, 100 U/mL penicillin and MycoZap^TM^ Prophylactic. This cell line was differentiated into foam cells after incubation with acetylated LDLs (LDLac). Briefly, J774A.1 cells were plated (10^5^ cells/well) in a 24 well-plate and 24 h later, 125 µg/mL LDLac was added to each well and the cultures were maintained for an additional 24 h.

Human vascular smooth muscle cells (VSMCs) were isolated from carotid arterial atherosclerotic tissue samples. Carotid atheroma plaque samples were obtained by carotid endarterectomy. Samples were placed on ice and processed immediately. An enzymatic tissue digestion method was used to isolate and culture VSMCs from atherosclerotic tissue samples in two consecutive digestions.

First, tissue was digested for 3 h at 5% CO_2_ and 37 °C with 300 U/mL of (Collagenase type I) followed by a second overnight digestion with 220 U/mL of ColI at 5% CO_2_ and 37 °C. Digested tissue was filtered by a 100 µm nylon Falcon™ Cell Strainer (CLS431752-50EA, Sigma-Aldrich, St Louis, MO, USA) to remove undigested tissue and then, cells were plated in selective medium (2 ng/mL FGFb, 20 ng/mL IGF-1 and 0.5 ng/mL EGF, 5 ng/mL Heparin, 5% NCS, 0.2 µg/mL BSA, 2 mM l-glutamin, 100 µg/mL streptomycin and 100 U/mL penicillin in Gibco’s Medium 231), which promotes selective VSMC growth. All the experiments were carried out with cells in passage zero at 70% confluence to reach a situation as close as possible to the real one.

When appropriate, cells were treated with T090, an LXR agonist, which induces ABCA1 expression.

This study was approved by the local ethical committee (Ethical Committee of Clinical Research, Basurto University Hospital. Project identification code: PI2018015; approval date: 3 November 2019; name of the committee: Basque Country Research Ethics Committee (CEIm-E)). All carotid atheroma plaques were collected from patients who had signed written informed consent. This research was performed in agreement with the principles outlined in the Declaration of Helsinki.

### 2.8. Cholesterol Efflux Assay

Functionality of rHDLs was analyzed through their capacity to induce cholesterol efflux from cells loaded with TopFluor-Cholesterol as described before [54]. Labelling medium was prepared by complexing a mixture of cholesterol and TopFluor-cholesterol (3:1, M:M) with β-cyclodextrin (16 mM). Once mixed, cholesterol was dried and mixed with an 80-molar excess of β-cyclodextrin dissolved in Minimum Essential Medium Eagle (MEM)—Hepes 25 mM media; pH 7.4. Finally, the mixture was sonicated at 40 °C in a water bath for 30 min to re-suspend cholesterol and further incubated for 3 h at 37 °C in agitation.

Differentiated cells were loaded with labelling media diluted in RPMI-1640 containing 2% of FBS, 4% BSA and 4 µg/mL ACAT inhibitor (1:1, *v:v*) for an hour. Cells were then washed and incubated in RPMI-1640 containing 2% BSA and 2 µg/mL ACAT inhibitor for 15 h with or without 3 µM of TO90 LXR agonist in order to induce ABCA1 upregulation [55].

After resting time, rHDLs were added to the cells in MEM-Hepes 25 mM (pH 7.4) containing 2 µg/mL ACAT inhibitor. The doses of rHDLs were defined by the quantity of human apoA-I present in the infusion. After 6 h incubation, media and cells were collected to assess cholesterol efflux. As an internal control of the experiment, FBS 20% and BSA 10 µg/mL were added to TopFluor-Cholesterol loaded cells to obtain maximum and non-specific efflux, respectively, in the absence of rHDL. Finally, media were collected and centrifuged for 15 min at 3800 rpm to remove cell debris. Cell monolayers were washed gently with MEM-Hepes 25 mM and solubilized with lysis buffer (50 mM Tris–HCl, pH 7.5, 0.1% SDS, 0.1% deoxycholic acid, 0.1 mM EDTA, 0.1 mM EGTA, 1% NP-40, 5.3 mM NaF and 1.5 mM NaP) during 30 min in a plate shaker at room temperature. Fluorescence of both culture media and cell lysates was measured using a Synergy™ HTX Multi-Mode microplate reader (λex: 485 ± 20 nm, λem: 528 ± 20 nm) and fluorescence intensities (*FI*) were used to calculate cholesterol efflux in each condition as follows:

$$cholesterol\;efflux\;\%\;=\;\frac{culture\;media\;FI}{culture\;media\;FI\;+\;cell\;lysate\;FI}\;\times \;100$$

Culture media *FI* was obtained by subtracting the fluorescence intensity of the media with no acceptors. Specific efflux of each acceptor was calculated by subtracting the efflux to BSA.

### 2.9. Statistical Analysis

All measurements were performed at least 3 times, unless otherwise stated, and results are presented as mean ± SD. A Shapiro–Wilk test was performed to confirm that the data were normally distributed. The null hypothesis was verified, indicating that the data were normally distributed. As the intention was to compare HDL with each rHDL composition or DPPC rHDL with other rHDLs, that is, comparison of 2 variables, Student *t*-tests were employed for analysis. A 2-tailed Student’s *t* test with a significance level of 0.05 was used to test differences in cholesterol efflux efficiency. All statistical analyzes were performed with the SPSS 25 (SPSS, Inc., Chicago, IL, USA).

## 3. Results

### 3.1. Development and Biophysical Characterization of rHDL

**Figure 1:**
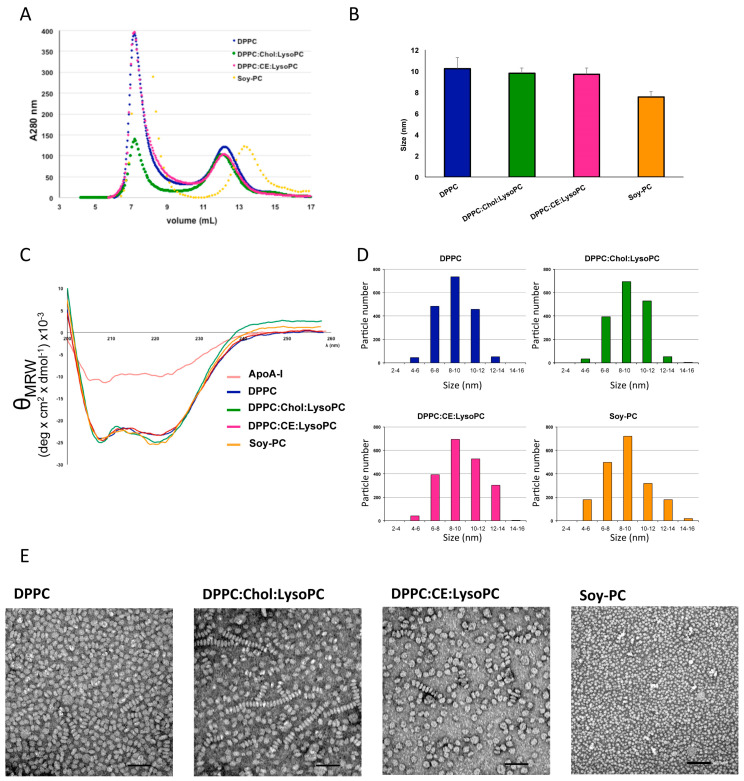
Development and biophysical characterization of rHDL. (**A**) Gel filtration profiles of rHDL reconstituted with different lipids and apoA-I. Nanoparticles were analyzed by gel filtration chromatography on a Superdex 200 column eluted at 4 °C. The profiles were monitored using absorbance at 280 nm. (**B**) rHDL size (hydrodynamic diameter) determination by dynamic light scattering (DLS). Each rHDL preparation present 9–10 nm diameter when analyzed trough DLS. No significant differences between different compositions. (**C**) Circular dichroism of rHDL preparations and apoA-I protein in solution at 25 °C in buffer TEN (pH 8). θ_MRW_: mean residue ellipticity. (**D**) Frequency histograms showing particle size distribution of rHDL determined from TEM images. (**E**) Representative rHDL transmission electron microscopy images. DPPC, DPPC:Chol:LysoPC (8.5:1:0.5), DPPC:CE:LysoPC (7.5:2:0.5) and Soy-PC. Magnification 100×. Scale bar of 100 nm. rHDL size distribution in (**D**) was measured as Feret diameter calculated from 1600 particles.

HDL were reconstituted with different phospholipid mixtures (Soy-PC, DPPC, DPPC:Chol:lysoPC (85:10:5 mol%) and DPPC:CE:lysoPC (75:20:5 mol%) as indicated in the Materials and Methods section. The reconstitution ratio of apoA-I:lipid was optimized to 1:125. The rHDLs, aggregates, and free apoA-I were detected and separated by size exclusion chromatography on a Superdex 200 column as shown in Figure 1A. When applying the rHDLs samples, the aggregates were present in the void volume of the size exclusion column at 7–9 mL, and a rHDL homogenous peak was centered at 11–13 mL, preceding free apoA-I at 15 mL (Figure 1A).

DLS was used to characterize rHDL size (hydrodynamic diameter) and homogeneity. The size distribution of nanodiscs indicated that rHDLs have an average diameter of ~10 nm (Figure 1B).

We next evaluated, by circular dicroism (CD) measurements, α-helical structure in the purified rHDL and apoA-I (Figure 1C). The higher α-helical content of rHDL shown by rHDL compared to free apoA-I (≈2.2–2.5 times, Table 1) indicates a correct protein conformation and well-structured protein within the nanodisc (Figure 1C).

Negative stain electron microscopy (NS-EM) was also used to qualitatively examine homogeneity of the rHDLs and to measure particle diameter (Figure 1D,E). The peak population of the selected 1600 particles was in the diameter range of 8–10 nm, confirming the values obtained by DLS (Figure 1D). The determined diameters for the rHDLs were: DPPC 9.0 ± 1.6, DPPC:Chol:lysoPC 9.2 ± 2.4, DPPC:CE:lysoPC 10.8 ± 2.2 and Soy-PC 8.7 ± 2.3. Mostly, all nanodiscs appeared as single particles oriented randomly on the staining grid (Figure 1E). The characteristic stacked nanoparticles were also observed by NS-EM but they appeared in a non-significant number. rHDL morphology was approximately circular, consistent with a discoidal shape (Figure 1E).

**Figure 2:**
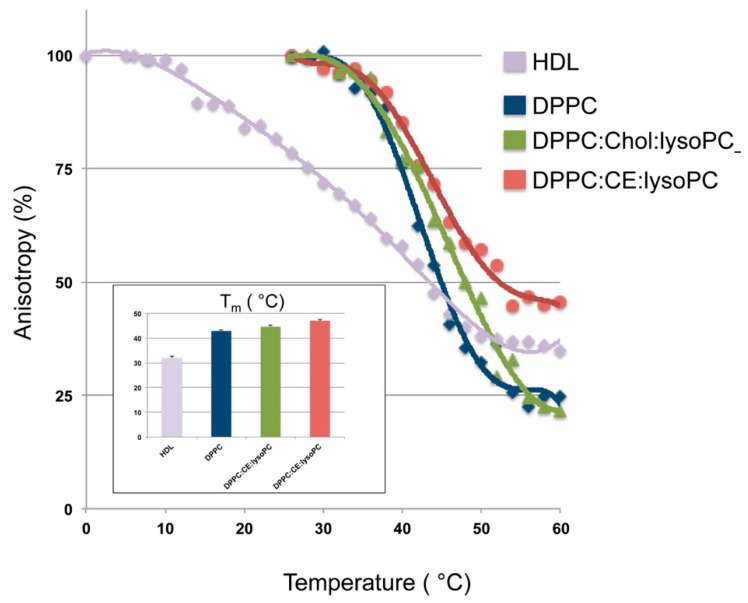
Fluorescence anisotropy of DPH in HDL, DPPC, DPPC:Chol:lysoPC and DPPC:CE:lysoPC rHDLs as a function of temperature. The inflection point of the plot gives the phase transition temperature of high-density lipoproteins (HDL) and rHDLs. Insert shows the phase transition temperatures. DPH was excited at 360 nm and fluorescence anisotropy was measured at 428 nm. All measurements were carried out in TEN buffer pH 8. Concentration of HDL and rHDL was kept constant at apoA-I 2 µM for all measurements. Data points shown are means ± S.D. of at least three independent measurements. DPH: 1,6-diphenyl-1,3,5-hexatriene.

Transition temperature of the rHDLs lipid moiety was assessed by steady state fluorescence anisotropy using DPH, which localizes to the hydrocarbon core of the lipid bilayer [56]. The temperature-dependent fluorescence anisotropy changes of DPH allows determining phase transition temperature of the different lipid mixtures in rHDLs [57]. As shown in Figure 2, the phase transition temperature of DPPC rHDL obtained from our measurement is 42.9 ± 0.3 °C, which is similar to the literature value-range of phase transition temperature of DPPC nanodiscs [58]. The addition of Chol/lysoPC or CE:lysoPC to the nanodiscs increases the phase transition temperature by 1.7 and 4.1 °C compared to DPPC alone, respectively (Figure 2). As shown in the Figure 2 inset, DPPC:Chol:lysoPC and DPPC:CE:lysoPC T_m_ are 44.6 ± 0.6 and 47.0 ± 0.5 °C, respectively. As expected due to its lipid composition, HDL T_m_ was 32.0 ± 0.3 °C in the range of the previously described transition temperature of lipoproteins (27–34 °C) [59]. Fluorescence anisotropy changes of DPH Soy-PC nanodisc were not assessed because they are already at liquid-crystalline state below 0 °C.

### 3.2. Effect of rHDL Lipid Composition on Cholesterol Efflux In Vitro

#### 3.2.1. Cholesterol Efflux Promoted in Human and Murine Macrophages

**Figure 3:**
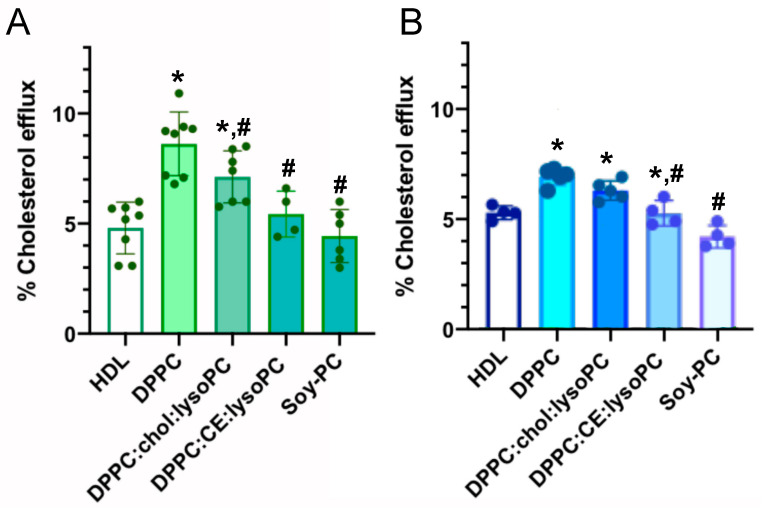
Effect of HDL, DPPC, DPPC:Chol:lysoPC, DPPC:CE:lysoPC and Soy-PC rHDLs on cholesterol efflux in (**A**) human THP-1 and (**B**) murine J774A.1 derived macrophages. rHDLs were added to the cells in MEM-Hepes 25 mM (pH 7.4) containing 2 µg/mL ACAT inhibitor and incubated during 6 h to promote cholesterol efflux. Cholesterol efflux was calculated as described in Methods. The data represent the means ± S.D. of at least three independent measurements. Levels of significance were determined by a two-tailed Student’s *t*-test. * *p* < 0.01 compared to HDL and # *p* < 0.01 compared to DPPC.

**Figure 4:**
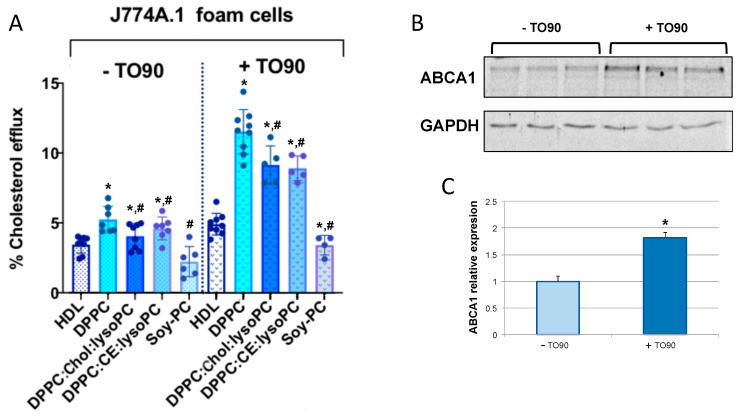
Effect of HDL, DPPC, DPPC:Chol:lysoPC, DPPC:CE:lysoPC and Soy-PC rHDLs on cholesterol efflux in (**A**) murine J774A.1 derived foam cells and murine J774A.1 derived foam cells stimulated with TO90. (**B**) ABCA1 levels in both TO90 stimulated and non-stimulated foam cells. (**C**) Expression levels of ABCA1 determined by optical density. rHDLs were added to the stimulated and non-stimulated cells in MEM-Hepes 25 mM (pH 7.4) containing 2 µg/mL ACAT inhibitor and incubated during 6 h to promote cholesterol efflux. Cholesterol efflux was calculated as described in Methods. The data in A and C, represent the means ± S.D. of at least three independent measurements. Figure 4B correspond to a representative western blot of *n* = 3. Levels of significance were determined by a two-tailed Student’s *t*-test. * *p* < 0.01 compared to HDL and # *p* < 0.01 compared to DPPC.

**Figure 5:**
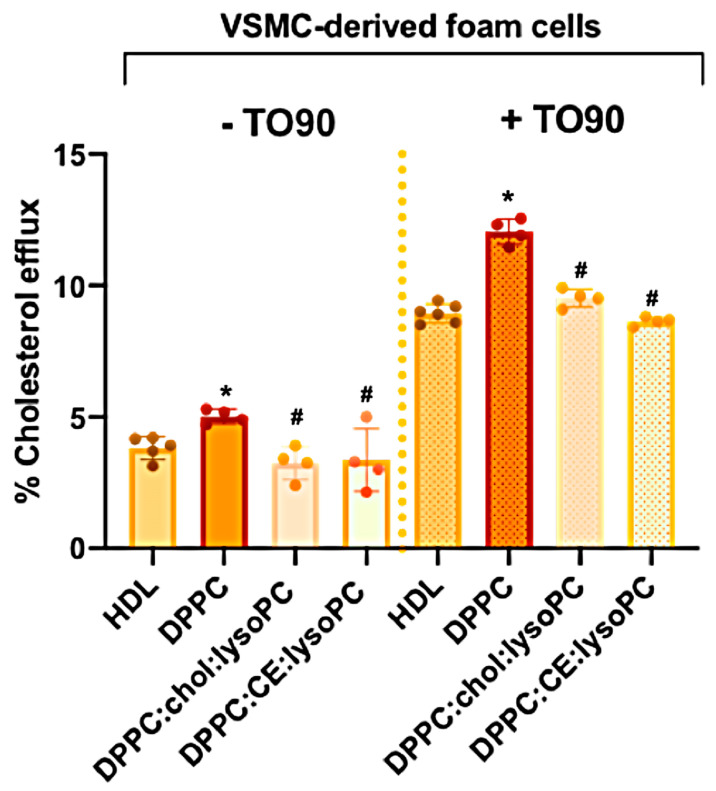
Effect of HDL, DPPC, DPPC:Chol:lysoPC and DPPC:CE:lysoPC rHDLs on cholesterol efflux in vascular smooth muscle cells (VSMC) derived foam cells stimulated or not with TO90. rHDLs were added to the stimulated and non-stimulated cells in MEM-Hepes 25 mM (pH 7.4) containing 2 µg/mL ACAT inhibitor and incubated during 6 h to promote cholesterol efflux. Cholesterol efflux was calculated as described in Methods. Data represent the means ± S.D. of at least three independent measurements. Levels of significance were determined by a two-tailed Student’s *t*-test. * *p* < 0.01 compared to HDL and # *p* < 0.01 compared to DPPC.

The effect of rHDL lipid composition on promoting cholesterol efflux was assessed both in human THP-1 and murine J774A.1 macrophages, and in human VSMC-derived foam cells (Figure 3, Figure 4 and Figure 5, respectively). Cells were loaded with TopFluor-cholesterol and cholesterol efflux was determined following incubation with rHDL of different lipid compositions.

As shown in Figure 3A, incubation of THP-1 derived macrophages with DPPC and DPPC:Chol:lysoPC rHDLs showed a significantly higher cholesterol efflux than those incubated with human HDL; in fact, DPPC rHDL particles were a 51% more efficient than HDLs and, DPPC:Chol:lysoPC rHDLs showed a 34% increased efficiency compared to HDLs. In contrast, DPPC:CE:lysoPC and Soy-PC rHDLs showed a similar cholesterol efflux compared to HDLs.

Similar results, but to a lesser extent, were obtained in J774A.1 macrophages. As shown in Figure 3B, DPPC and DPPC:Chol:lysoPC rHDLs induced a significantly higher cholesterol efflux from the cells than those incubated with human HDL; in this case, DPPC rHDL particles were 35% more efficient than HDLs and, DPPC:Chol:lysoPC rHDLs showed increased efficiency by 24% compared to HDLs. In contrast, DPPC:CE:lysoPC and Soy-PC rHDLs showed similar cholesterol efflux compared to HDLs.

Cholesterol efflux induced by rHDLs in J774A.1 macrophage-derived foam cells showed similar results to those determined in THP-1 and J774A.1 cells (Figure 4A). Incubation with DPPC rHDL induced a significantly higher cholesterol efflux when compared with HDL. Although the cholesterol efflux induced by HDL was lower when compared to non-foam J774A.1 cells, the efficiency of DPPC nanodiscs resulted in a higher efficiency when compared to that determined with DPPC rHDL in non-foam cells (57% vs. 35%, respectively). DPPC:Chol:lysoPC, DPPC:CE:lysoPC also showed a higher cholesterol efflux compared to HDL (≈20%, ≈25%, respectively) while Soy-PC rHDLs showed a similar cholesterol efflux than that determined for HDL. Next, the effect of ABCA1 overexpression on cholesterol efflux induced by rHDLs was examined. *Abca1* mRNA expression was stimulated by incubating J774A.1 foam cells with TO90 and cholesterol efflux was determined in similar conditions as before. As shown in Figure 4A, upregulation of the ABCA1 transporter significantly enhanced the cholesterol efflux induced by rHDL, with the cholesterol efflux induced by DPPC being 140% more effective than that induced by HDL. The effect of DPPC:Chol:lysoPC and DPPC:CE:lysoPC rHDLs on cholesterol efflux was 100% higher than HDL. On the other hand, Soy-PC rHDL induced cholesterol efflux was similar to that induced by HDL. As shown in Figure 4B,C, TO90 almost induced twice the upregulation of ABCA1 transporter, indicating that cholesterol efflux induced by DPPC, DPPC:Chol:lysoPC and DPPC:CE:lysoPC rHDLs is efficiently enhanced by upregulating the transporter (Figure 4).

#### 3.2.2. Cholesterol Efflux Promoted in Human VSMC-Foam Cells

VSMCs extracted from carotid arterial atherosclerotic tissue samples showing foam cell phenotype obtained from carotid endarterectomy were used to determine the ability of rHDL to induce cholesterol efflux [60]. As shown in Figure 5, upon incubation with rHDLs, only DPPC nanodiscs induced a slight but significant increase in cholesterol efflux (22%) when compared with HDL. DPPC:Chol:lysoPC and DPPC:CE:lysoPC rHDLs showed similar cholesterol efflux to HDL. Upregulation of ABCA1 in VSMC by TO90 increases cholesterol efflux to HDL significantly when compared to non-stimulated cells (Figure 5). In addition, cholesterol efflux to DPPC rHDL was also significantly increased compared to HDL upon TO90 treatment (Figure 5). The effect of DPPC:Chol:lysoPC and DPPC:CE:lysoPCrHDLs in TO90 stimulated cells was similar to HDL (Figure 5).

## 4. Discussion

Reverse cholesterol transport from peripheral tissues to the liver is a major atheroprotective event, with cholesterol efflux as a rate-limiting step [61,62]. Two principal transporters contribute to this process: ABCA1 and ABCG1 [63]. ABCA1 results in the formation of discoidal HDL, while ABGC1 mediates cholesterol efflux through a diffusion mechanism that increases the pool of active cholesterol available for efflux [64].

Although the exact mechanisms of cholesterol efflux mediated by the ABCA1 transporter are not known, a central feature of cholesterol transfer is apoA-I interaction with ABCA1, which stabilizes the transporter and induces bending of the plasma membrane bilayer. This process creates a high curvature site that allows apoA-I to solubilize lipids by binding to exovesiculated plasma membrane domains [18,65]. Although the structural and physical features of apoA-I variants and mimetic peptides that are important in the formation of HDL-like particles have been previously investigated [66,67,68,69], the effect of the lipid content of rHDL has been less well characterized. Therefore, in this study, we sought to explore the effect of the lipid composition of rHDL on cholesterol efflux in macrophages, macrophage-derived foam cells unstimulated or stimulated with TO90 and foam-VSMC unstimulated or stimulated with TO90 to upregulate ABCA1 expression. Efficiency of cholesterol efflux mediated by DPPC, DPPC:Chol:LysoPC or DPPC:CE:LysoPC and Soy-PC rHDLs with similar sizes has been assessed. Here, we have used three lipid compositions resembling different maturation stages of natural HDLs in vivo and Soy-PC, which is the major lipid composition constituent used in rHDL that are being tested in clinical trials [38]. The rationale of this study relies on the information provided by previous studies using rHDLs with different lipid mixtures, which has already indicated that lipid composition plays a significant role in cholesterol efflux from macrophages [70]. According to the results obtained in this work, DPPC rHDLs, mimicking nascent HDL are the most effective particles in inducing cholesterol efflux in all the cellular models used. When compared to HDL-induced cholesterol efflux, DPPC rHDLs were 20–40% more efficient depending on the cell culture tested (Figure 3, Figure 4 and Figure 5). This effect can be attributed to the homogeneous composition of the DPPC rHDL as it has been previously shown that the phospholipid composition of HDL plays an important role in ABCA1-mediated cholesterol efflux and that enrichment of HDL with PC favors cholesterol efflux, in particular [71,72]. In addition, upregulation of ABCA1 with TO90 favored the cholesterol efflux induced by the nanoparticles, especially in macrophage derived foam cells (Figure 4).

Very interestingly, DPPC rHDLs also resulted in more efficiently favoring cholesterol efflux than Soy-PC, the lipid composition used in CSL-111 rHDLs [41,42] and in the smaller CSL-112 nanoparticles [23] currently being tested in humans. It has been described that rHDLs composed of saturated lipids exhibit greater cholesterol efflux from macrophages in vitro and cholesterol mobilization in vivo [73,74]. The more efficient cholesterol-efflux activity observed here with DPPC rHDL compared to Soy-PC rHDLs, can also be ascribed to the properties given by the different lipid composition of the nanodiscs. The main difference between Soy-PC and DPPC rHDL is that in the former, the existing 80% of PCs consist of a mixture of unsaturated fatty acids (C18:1, C18:2 and C18:3) and lyso-PC at 2.8% while composition in the latter is 100% DPPC. According to previously described data [75,76], the increased cholesterol efflux induced by DPPC rHDLs may be attributed to the higher physical binding affinity to cholesterol of saturated phospholipids compared with Soy-PC, in which the majority of phospholipids are unsaturated. Similarly, and in agreement with this, the different lipid composition of *apoA-I Milano* rHDL (ETC-216) and ETC-642 rHDL could explain the differences among the cholesterol efflux induced by the particles because they are constituted by POPC (1-palmitoyl-2-oleoyl-sn-glycero-3-phosphocholine) or a mixture of DPPC and sphingomyelin, respectively [43,77]. In addition, differences in the protein moiety may also contribute. It has already been shown that saturated long-chain-length phospholipids such as DPPC have higher physical binding affinity to cholesterol than POPC [75]. Additionally, the rigidifying effect of sphingomyelin present in ETC-642 rHDL can modify the physical properties of rHDLs resulting in a lower surface tension that could reduce the cholesterol exchange efficiency between membranes [78].

One of the goals of this work was to study the cholesterol efflux efficiency among rHDL which simulate different maturation stages. To do so, we have compared the effects of DPPC rHDL, with DPPC:Chol:lysoPC and DPPC:CE:lysoPC that can be considered particles in a more mature stage. DPPC:Chol:lysoPC rHDL resembling nascent HDLs having incorporated free cholesterol, and DPPC:CE:lysoPC resembling those in which cholesterol is esterified by the effect of Lecithin–cholesterol acyltransferase (LCAT). As shown in Figure 3, Figure 4 and Figure 5, DPPC rHDL were the most efficient particles in inducing cholesterol efflux, an effect that may be mediated by the physicochemical characteristics of the nanoparticles. The role of membrane lipid composition in cholesterol exchange between membranes is not well understood, however phospholipids and fatty acyl chains have been shown to influence the rate of cholesterol movement between membranes [79,80,81]. In addition, curvature of the lipid bilayer that is imposed by the overall geometry of lipids shows a physiological significance in cholesterol transfer [82]. Lipid geometry is defined by the ratio between the size of the polar head group and acyl chain saturation. PC is a cylindrical lipid that forms flat monolayers [83]. Conversely, the large head group to acyl chain ratio in lyso-PC confers an inverted conical shape to the lipids, thereby favoring a positive curvature of the membrane by bending the monolayer away from the head groups [84,85,86]. Addition of cholesterol increases the packing of conical lipids (such as lyso-PC), and thus disfavors spontaneous curvature induced by the lysophosphospholipid [87] while the esterification of the 3′ hydroxyl group of cholesteryl esters is structurally consistent with substantially increased positive curvature [88]. Recent studies have shown that membrane curvature is an active driving force in many processes involving membrane remodelling and cholesterol exchange [89]. It has been shown that cholesterol transfer is about 10 times faster from donor bilayers with high positive curvatures and when the acceptor bilayer is planar [82,90]. Although the curvature of biological membranes is very low, the bending of the plasma membrane bilayer by ABCA1 creates a high curvature that can facilitate cholesterol transfer [18,65]. The cholesterol transfer will be favored thermodynamically from the high curvature promoted by ABCA1 in the cell membrane to DPPC rHDLs instead of DPPC:Chol:lysoPC and DPPC:CE:lysoPC, because the former are planar bilayers and the latter two have positive curvatures [91].

The transfer of cholesterol between membranes is strongly dependent on temperature and is affected by the lipid composition, suggesting that membrane fluidity strongly influences the transfer rate [79]. In this work we have also assessed of thermotropic phase transition of HDL and rHDLs to compare their fluidity. Attending to the T_m_ of DPPC, DPPC:Chol:lysoPC and DPPC:CE:lysoPC rHDLs, the nanoparticles are more rigid as Chol, and CE are incorporated, DPPC < DPPC:Chol:lysoPC < DPPC:CE:lysoPC, showing a T_m_ increment of 1.7 and 4.1 °C compared to DPPC alone, respectively. This effect could indicate that particles with high transition temperatures could be less efficient in accommodating cholesterol from the plasma membrane due the intrinsic physical characteristics of the rHDL bilayer and that the higher T_m_ of rHDL, the lower the cholesterol efflux rate induced by the rHDL. However, attending to this hypothesis, Soy-PC would be the most efficient particles inducing cholesterol efflux followed by HDL, which show Tm below 0 °C and 32.0 ± 0.3 °C, respectively. The lower capacity of promoting cholesterol efflux shown by HDL and Soy-PC indicates that rather than Tm, lipid composition favoring higher binding affinity of cholesterol (saturated acyl chains) and planar bilayer geometries such as shown by DPPC rHDLs are more favorable to promote cholesterol efflux.

## 5. Conclusions

In this work, the effect of the lipid composition of rHDL on cholesterol efflux in several cell models has been characterized to determine optimal parameters to achieve maximal cholesterol efflux rates. Three different lipid mixtures were used, mimicking different maturation stages of natural HDLs in vivo and Soy-PC, which is the lipid composition constituent used in the rHDL in clinical trials. Our results indicate that DPPC rHDLs, which resemble nascent HDL, are the most effective particles inducing cholesterol efflux in all the cellular models used. Among the mechanisms underlying their effects are: (1) the higher physical binding affinity of cholesterol to saturated long-chain-length phospholipids in pure DPPC rHDLs and, (2) geometry of lipids within a lipid bilayer influences the rate of cholesterol movement between membranes, and is favored when the donor bilayer has a high positive curvature and the acceptor bilayer is planar, as occurs with DPPC rHDLs.

In sum, the results presented here indicate that rHDLs with a lipid composition similar to nascent HDLs, are more efficient in promoting cholesterol efflux, and their physical characteristics should be taken into consideration to design more efficient rHDL to be used as a cholesterol efflux promoting nanodisc. In addition to promoting cholesterol efflux from cells and taking advantage of the biophysical features of the nanoparticles used in this study, functionalized rHDL could be used to remove the extracellular accumulation of cholesterol in lesions, thus constituting a potential therapeutical tool to avoid plaque progression.

## Figures and Tables

**Table 1:** α-helical content of apoA-I and rHDL determined by CD.

	α-Helical Content	α-Helicity Ratio rHDL/apoA-I
ApoA-I	30.7 ± 2.3	-
DPPC	70.0 ± 2.8 *	2.3 ± 0.3 *
DPPC:Chol:LysoPC	77.4 ± 6.6 *	2.5 ± 0.02 *
DPPC:CE:LysoPC	64.7 ± 3.8 *	2.2 ± 0.04 *
Soy-PC	70.3 ± 3.5 *	2.5 ± 0.3 *

α-helical content calculated from ellipticity values at 222 nm. R is the ratio of alpha helicities between nanoparticles and free protein. Once incorporated into rHDL, apoA-I increases its helicoidal structure 2 times in each composition of rHDL, with no differences among them. Data represent the mean ± S.D. (*n* = 3). All measurements were performed independently 3 times and levels of significance were determined by a two-tailed Student’s *t*-test. * *p* < 0.01 compared to apoA-I. rHDL: reconstituted HDL; DPPC: 1,2-dipalmitoyl-sn-glycero-3-phosphocholine; LysoPC: 1-palmitoyl-2-hydroxy-sn-glycero-3-phosphocholine.
